# Non-vascularised fibula grafts for reconstruction of segmental and hemicortical bone defects following meta- /diaphyseal tumour resection at the extremities

**DOI:** 10.1186/s12891-017-1640-z

**Published:** 2017-07-05

**Authors:** Ulrich Lenze, Stefanie Kasal, Fritz Hefti, Andreas Heinrich Krieg

**Affiliations:** Department of Orthopaedics, University Children’s Hospital of both Basel (UKBB), Spitalstrasse 33, CH-4056 Basel, Switzerland

**Keywords:** Bone defects, Bone tumour, Sarcoma, Non-vascularised fibula, Defect reconstruction, Autograft

## Abstract

**Background:**

The reconstruction of meta−/diaphyseal bone defects following bone tumour resection is challenging, and biological treatment options should be applied whenever possible, especially in benign lesions and early stage sarcomas. We aimed to evaluate the results of segmental (SR) and hemicortical reconstructions (HR) at the extremities using non-vascularised fibula grafts.

**Methods:**

We retrospectively enrolled 36 patients who were treated with non-vascularised fibula reconstructions (15 SR, 21 HR) after bone tumour resection (15 malignant, 21 benign). All cases were evaluated regarding consolidation, hypertrophy at the graft-host junctions, and complications; moreover, the functional and oncological results were assessed. The mean follow-up was 8.3 years (2.1–26.6 years).

**Results:**

Primary union was achieved in 94% (SR 87%, HR 100%) of patients, and 85% (SR 81%, HR 88%) showed hypertrophy at the graft-host junction. The overall complication rate was 36% with 4 patients (11%) developing local recurrence. There was a significant correlation between the development of mechanical complications (fracture, delayed-/non-union) and a defect size of ≥12 cm (*p* = 0.013), segmental defects (*p* = 0.013) and additional required treatment (*p* = 0.008). The functional outcome was highly satisfactory (mean MSTS score 86%).

**Conclusions:**

Due to encouraging results and advantages (such as their remodelling capacity at the donor site), non-vascularised fibula reconstructions should be considered a valuable alternative treatment option for patients with hemicortical defects or segmental reconstructions of less than 12 cm in which no additional neo-/adjuvant treatment is necessary.

## Background

Bone tumours in the meta-/diaphyseal region of long bones are rare (<10%), and the reconstruction of emerging bone defects (segmental or hemicortical) are therefore challenging; however, the best treatment method has been unclear until recently [1, 2].

Modular intercalary tumour endoprostheses are frequently used due to their free availability, high cost effectiveness and quick recovery time, but the reported complication rates of these endoprostheses are sometimes high [2, 3]. Therefore, given that a considerable number of patients with malignant primary bone tumours have been cured due to interdisciplinary treatment strategies, biological reconstructions should be applied whenever possible. However, most specialists agree that biological reconstructions should especially be used in patients with stage I and, if useful, stage II tumours, whereas in patients with advanced primary bone tumours (stage III) or secondary lesions (metastases)—in which early full weight bearing and functionality are major concerns compared to durability—tumour endoprostheses are preferred [4, 5].

Depending on the localization, defect size and shape (segmental/hemicortical), underlying entities and adjuvant treatment modalities, biological reconstruction strategies include massive or hemicortical allografts (with or without vascularised autografts) [6–8], distraction osteogenesis [9], replantation of the sterilized tumour-bearing bone segment (e.g. after extracorporeal irradiation) [10], the induced membrane technique [11] or the use of vascularised or non-vascularised bone grafts [1, 12, 13].

The use of non-vascularised fibula grafts originated at the beginning of the twentieth century and was the gold standard for biological reconstructions for more than 60 years. Advantages of this method compared to the use of vascularised autografts consist of the remodelling capacity at the donor site, an easier operative technique and a shorter operative time [13, 14]. However, since non-vascularised fibula grafts are thought to lack biological activity and have a high risk of resorption, vascularised fibula grafts have been more frequently used for defect reconstructions during the last 40 years [1, 12, 15]. To date, there are a few reports on defect reconstructions after bone tumour resection using non-vascularised fibulae, but most of these studies did not focus on the extremities or have a rather small cohort of patients [13, 16–20].

We present the largest case series on non-vascularised fibula reconstructions at the extremities following tumour resection (segmental and hemicortical). The aim of this retrospective study was to analyse the results with respect to variables such as consolidation, hypertrophy at the graft-host junctions, and complications as well as assess the functional and oncological outcomes.

## Methods

**Fig. 1:**
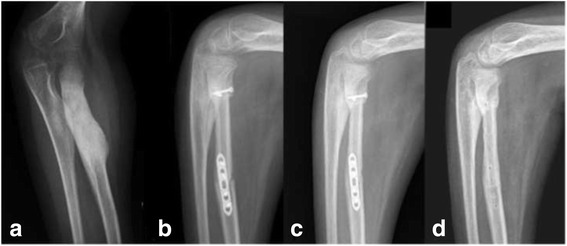
Extended osteoid osteoma of the left proximal radius in a 9-year-old male patient **a** Postoperative conventional X-rays 3 months after segmental resection **b** The single strut was fully integrated 5 months after surgery and exhibits hypertrophy at its junctions **c** Plate removal was performed 7 months after the initial surgery **d**

**Fig. 2:**
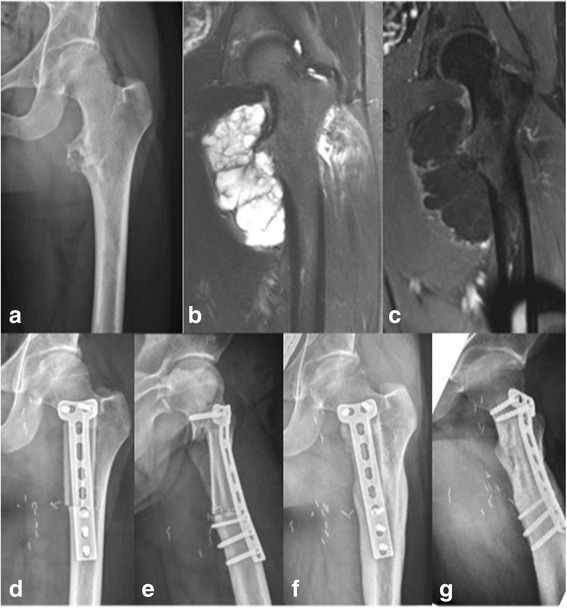
Preoperative imaging of a 28-year-old male patient with periosteal chondrosarcoma (G2) of the left proximal femur: conventional X-rays **a** STIR MRI sequence **b** e-Thrive MRI sequence with contrast agent **c**. Postoperative conventional antero-posterior **d** and latero-lateral **e** X-rays following wide resection and hemicortical reconstruction with two non-vascularised fibula struts. Complete integration of both struts and remodelling of the resected segment on conventional X-rays was observed 10 months after surgery **f**, **g**

**Fig. 3:**
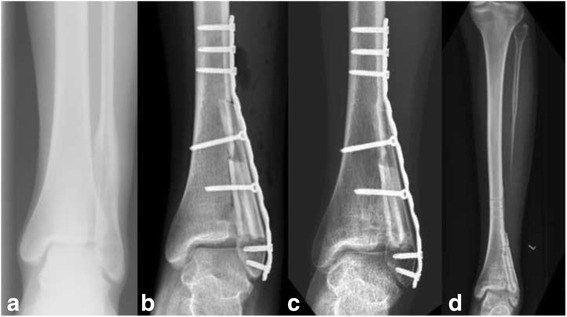
Ewing’s sarcoma of the distal fibula in a 15-year-old female national squad triathlete **a** After wide resection of the tumour under preservation of the malleolar tip, the distal fibula was reconstructed with a non-vascularised fibula strut by performing a tibio-fibular synostosis **b** After complete integration and bony consolidation of the fibula graft **c** removal of the plate as well as the screws was planned as the patient felt bothered during sports activities **d** Partial remodelling of the fibula with ossifications along the periosteum was seen on conventional X-rays of this patient **d**

We retrospectively enrolled 36 patients (20 male, 16 female) with bone tumours at the extremities (15 malignant, 21 benign) and a mean age of 24 years (range 6–68 years) who were treated with non-vascularised fibula reconstructions between 1976 and 2012 at our institution (Table 1). Segmental reconstructions (SR) (Fig. 1) were performed in 15 patients (42%) and hemicortical reconstructions (HR) (Fig. 2) in 21 patients (58%). In total, 9 chondrosarcomas (25%), 5 aneurysmal bone cysts (14%), 3 osteochondromas (8%), 2 osteosarcomas (6%), 2 Ewing’s sarcomas (6%), and 15 “others” (41%) were included (Table 1). Affected sites were the humerus (6), radius (2) (Fig. 1), ulna (1), femur (20) (Fig. 2), tibia (5) and fibula (2) (Fig. 3). The last two cases had disease located in the proximal (case 8) and distal regions (case 25) of the fibula. In one patient (case 8), who was operated on more than 25 years ago, a non-vascularised fibula graft from the contralateral side was transplanted to allow the re-insertion of the biceps tendon as well as the lateral collateral ligament. In the second patient, who was a national squad triathlete, an ipsilateral fibula graft was used to reconstruct the ankle joint by performing a tibio-fibular synostosis (Fig. 3). All operations were performed by the two senior authors (FH, AHK).

**Table 1:** Patient characteristics and results

Case	Diagnosis	Age (years)	Site	Defect length (cm)	Reconstruction	Neo−/adjuvant therapy	Time to union (weeks)	Functional score^b^ (%)	Fibular remodelling	Complications		
										Fatigue fracture	Infection	Others
1	Hemangiopericytoma	5.5	Femur	12	s	NAC	Non-union	80	complete	1		
2	Aneurysmal bone cyst	11.0	Femur	7	h		12	90	complete			
3	Chondrosarcoma	13.6	Femur	14	s		32	90	partial			PP (transient)
4	Osteosarcoma	14.0	Tibia	12	s	NAC, AC	61^a^	70	complete			
5	Chondrosarcoma	16.1	Humerus	20	s	AC	29	80	complete	1		
6	Fibrous dysplasia	16.8	Humerus	22	s		25	83	NE			LR
7	Adamantinoma	17.2	Tibia	24	h		45	80	partial			
8	Giant cell tumor	17.7	Fibula	6,5	s		11	90	NE			
9	Osteochondroma	20.9	Femur	9,5	h		31	67	NE			
10	Chondrosarcoma	23.3	Femur	13	s		38	67	partial			
11	Fibrous dysplasia	24.2	Radius	19	s		24	80	NE			
12	Ewing’s sarcoma	26.5	Humerus	14	s	NAC, AC, AR	Non-union	37	NE		1 (deep)	
13	Chondrosarcoma	28.4	Femur	9	s		22	63	NE			
14	Chondrosarcoma	29.2	Humerus	10	s		18	90	NE	1		
15	Aneurysmal bone cyst	29.5	Humerus	14	h		26	90	none			
16	Chondrosarcoma	31.7	Femur	8	s		23	67	partial			
17	Chondrosarcoma	33.2	Femur	7	h		16	90	NE		1 (superficial)	LR PP (transient)
18	Osteochondroma	38.3	Femur	12	h		26	70	none			
19	Myxoid liposarcoma	44.6	Femur	9	h	AR	17	90	none			
20	Osteosarcoma	18.6	Femur	9	h		19	93	complete			LR
21	Osteofibrous dysplasia like adamantinoma	17.6	Tibia	7	h		18	93	complete			
22	Osseous hemangioma	33.7	Femur	18	h		14	87	partial	1		
23	Periosteal desmoid	68.3	Ulna	8	h		27	87	NE			
24	Chondrosarcoma	13.1	Femur	12	s		22	97	complete	1		
25	Ewing‘s sarcoma	16.2	Fibula	8	h	NAC, AC, AR	18	100	partial			
26	Osteofibrous dysplasia	14.6	Tibia	17	h		47	80	complete			
27	Synovial sarcoma	45.7	Femur	15	s		22	100	partial			
28	Giant osteochondroma	22.2	Femur	10	h		20	90	NE			
29	Osteoid osteoma	9.4	Radius	5	s		13	87	complete			
30	Giant cell tumor	38.8	Femur	7	h		26	93	partial			
31	Aneurysmal bone cyst	9.0	Femur	6,5	h		17	97	complete			
32	Aneurysmal bone cyst	42.9	Tibia	5	h		17	97	none			
33	Atypical periosteal chondroma	14.9	Humerus	5	h		27	97	partial			
34	Chondroblastoma	18.0	Femur	5,5	h		8	-	NE			
35	Aneurysmal bone cyst	5.6	Femur	6,5	h		7	93	complete			
36	Chondrosarcoma	28.2	Femur	8	h		19	83	partial			LR

Legend: *s* segmental, *h* hemicortical *NAC* neoadjuvant chemotherapy, *AC* adjuvant chemotherapy, *AR* adjuvant radiation therapy; *NE* not examined; *LR* local recurrence; *PP* peroneal nerve palsy

^a^delayed union; ^b^according to the Musculoskeletal Tumor Society Rating Scale (MSTS) [22]

In total, 18 of the 36 fibula reconstructions were evaluated in a previous study [13] but were included to evaluate the long-term effects of this technique.

Grafts were harvested using a posterolateral approach with preservation of the periosteum. At least 4 to 5 cm of the fibula were proximally preserved to avoid peroneal nerve injuries as well as maintain knee stability. Distally, a minimum fibula length of 8 to 10 cm was preserved to reduce the risk of instability of the ankle joint. Adjusting screws were not used at either the proximal or the distal region of the remaining fibula. At the host site, the non-vascularised fibula grafts were fixed with screws or wedged (press-fit) into the bone. In 17 patients, a plate fixation was implemented. Either single (21), double (11) or triple strut (4) reconstructions were performed depending on the defect size, shape and location. At the lower leg, all but two reconstructions (1 tibia, 1 fibula) were a single strut reconstruction, and at the femur, either double or triple strut reconstructions were performed in all the patients.

After surgery, patients were regularly followed every 6 to 12 weeks until consolidation was achieved. Conventional radiographs were taken to evaluate evidence of bony consolidation, hypertrophy, recurrence and complications. In 25 cases, an additional radiograph of the donor site was taken to evaluate the remodelling of the remaining fibula, which was classified as complete, partial or non-existent. Complete remodelling was assumed in all cases in which a solid bony bridge between the two ends of the persisting fibula with a similar or equal diameter was achieved. Cases with (incomplete) ossifications along the periosteum and/or a smaller diameter were categorized as partial remodelling.

Biological activity at the graft-host junctions was assumed in cases with hypertrophy (increase of diameter) in this area. Therefore, the hypertrophy index was calculated for all graft-host junctions as previously described by De Boer and Wood [21]. Since the third graft in the 4 cases with triple strut reconstructions (8 junctions) could not be evaluated regarding hypertrophy, a total of 102 of the 110 junctions were analysed for this variable. A hypertrophy index of more than 20% was considered significant, and an index between 0 and 20% was defined as biological activity at the graft-host junction without significant hypertrophy. An index of 0% or lower indicated a lack of biological activity or atrophy.

The functional outcome was determined according to the Musculoskeletal Tumor Society Rating Scale (MSTS) [22]. A score of 86% - 100% was assumed to be an excellent functional result, 70–85% as good, 50–69% as satisfactory and 0–49% as poor.

One patient for whom no functional results have been recorded was excluded from the functional analysis. The implemented fixation method as well as any donor/host site complications (such as fatigue fractures, infections, non-unions, etc.), additional treatments (chemotherapy, radiotherapy) and local recurrences were recorded.

### Statistical analysis

Metric data were described using the arithmetic mean as well as the maximum and minimum values. Frequencies of the nominal variables were indicated as percentages. Pearson or Spearman correlation coefficients were used to describe the relationship of the metric data. The median test was performed to compare medians of different groups (e.g. lower vs. upper extremity, fixation: plate vs. screw/press fit). Fisher’s exact test was used to identify significant relationships between nominal variables (tumour localization: upper vs. lower limb, defect size: ≥/< 12 cm, defect type: segmental vs. hemicortical, additional treatment, fixation method: plate vs. screw/press fit). Additionally, a multivariable logistic backward regression model was applied to assess complications such as fatigue fracture, non-union, delayed union, infection as well as relapse (predictor variables: fixation method, age, localisation, defect length, additional treatment, defect type), and a multivariable linear backward regression model was used to evaluate hypertrophy and consolidation time (predictor variables: defect size, age, neo−/adjuvant therapy, localisation, fixation method, defect type, defect length). A *p*-value <0.05 was considered statistically significant. Statistical analysis was performed with the SPSS software version 22 (SPSS Inc., Chicago, Illinois).

## Results

The mean defect size after tumour resection was 11 cm (range 5–24 cm, SD 5 cm), and the mean length of the harvested grafts was 16 cm (range 6.5–30 cm, SD 6 cm). In total, 6 patients received neo-/adjuvant treatment (Table 1). The average follow-up period was 8.3 years (range 2.1–26.6 years) and none of the patients were lost during follow-up.

### Hypertrophy and biological activity

Significant hypertrophy (>20%) was observed in 52% (SR 58%, HR 48%) of the evaluable graft-host junctions (102); hypertrophy of 20% or less was observed in 33% (SR 23%, HR 40%). In 15% (SR 20%, HR 11%) of the analysed graft-host junctions, neither hypertrophy nor atrophy was seen. Multivariable regression analysis revealed no significant influence of the patients’ age (*p* = 0.21), tumour localization (*p* = 0.38), defect size (*p* = 0.46), defect type (*p* = 0.35), additional treatment (*p* = 0.11) or fixation method (*p* = 0.28) on the hypertrophy rate (calculated as the mean hypertrophy rate per person).

### Consolidation

Primary union (<12 months) was seen in 94% of the 110 evaluated junctions (SR 87%, HR 100%), delayed union (>12 month) in 2% (SR 4%, HR 0%), and non-union in 4% (SR 9%, HR 0%). All instances of non-unions and delayed unions occurred in patients with segmental reconstructions. One patient with a non-union (case 1) healed after re-osteosynthesis and bone grafting with autologous cancellous bone. In the other patient (case 12), the fibula graft had to be removed due to an infected pseudarthrosis (this patient refused further treatment and lives with a spacer).

The mean time to primary union was 22 weeks (7–47 weeks, SD 9 weeks). Using multivariable linear backward regression analysis, age (*p* = 0.61), localization (*p* = 0.67), defect type (*p* = 0.35) or fixation method (*p* = 0.23) did not exert a significant influence on the union time. However, the defect size significantly influenced the consolidation time (*p* < 0.001, partial R^2^ = 0.35). The administration of neo-/adjuvant therapy did not significantly influence the union time (*p* = 0.58), but the univariate analysis revealed a statistically significant association between additional treatment and delayed-/non-union (*p* = 0.003).

### Fixation method

A screw and/or press-fit fixation was performed in 54% (19/36) of the patients and a plate fixation in the remaining 46% (17/36). Fibula grafts, which were fixed using a plate, tended to require a longer time until consolidation. Thus, the average union time was 27 weeks after plate fixation and only 20 weeks after screw and/or press-fit fixation. However, this difference was not statistically significant (*p* = 0.234).

### Functional results

The functional outcome was evaluated in 34 patients. The mean MSTS score was 86% (37–100%, SD 13%), and all but one patient had a score higher than 60%. Good or excellent results were seen in 86% (31/36), with only five patients exhibiting inferior results. There was no statistically significant correlation between the achieved MSTS score and either age (*p* = 0,981), the affected limb (lower vs. upper extremity, *p* = 0,217) or the fixation method (plate vs. screw/press fit, *p* = 0,146).

### Complications

The overall complications rate of our patients was 36% (*n* = 13), of which 10 patients (77%) required revision surgery. Among patients who were included into our previous study, no additional complications were recorded thereafter.

### Host site complications

In total, 2 infections (1 superficial, 1 deep infection) were recorded (6%), and 4 patients (11%) developed local recurrence. Fatigue fractures occurred in 5 of the 55 grafts (9%) among 5 different patients (14%). The initial stabilization method, which was not a statistically significant factor for the occurrence of complications, was a plate fixation in 2 patients (cases 22 and 24) and a screw fixation in 3 patients (cases 1, 5, and 14). One patient was conservatively treated (case 14), whereas the other four underwent revision surgery with re-osteosynthesis (cases 1, 5, 22, and 24). Four of the 5 fractures occurred in bone defects of 12 cm or greater which was a statistically significant factor (*p* = 0.013) for the occurrence of mechanical complications (fatigue fracture, delayed-/non-union), as shown by univariate analysis. Additionally, in patients with segmental reconstructions (*p* = 0.013) or who underwent adjuvant therapy (*p* = 0.006) a significantly higher mechanical complication rate was observed. Using multivariable logistic regression analysis, no statistically significant risk factors for development of complications (fatigue fracture, delayed-/non-union, infection, relapse, *p* ≥ 0.076) could be identified.

### Donor site complications

Aside from 2 patients with transient peroneal nerve palsy, no complications were recorded at the donor site. All patients were pain free and had cosmetically excellent results. There were no instances of tibial fracture, ankle joint instability or restriction in the range of motion of the knee or ankle joint at the last follow-up. Complete remodelling of the fibula was observed in 11 patients (44% of the 25 analysed patients) after a mean of 100 days (range 99–110 days); over the same duration, partial remodelling was observed in 10 patients (40%), and no remodelling was seen in the remaining 4 patients (16%).

## Discussion

This study was an analysis of outcome for patients undergoing non-vascularised fibula reconstructions (segmental and hemicortical) following tumour resection at the extremities with respect to consolidation, hypertrophy at the graft-host junctions, complications and functional outcome. Therefore, we retrospectively evaluated 36 patients with bone tumours (malignant *n* = 15, benign *n* = 21) at the extremities (upper extremity *n* = 9, lower extremity *n* = 27) who were treated with non-vascularised fibula reconstructions. Primary union of the graft-host junctions was recorded in 94% of the patients (SR 87%, HR 100%) after a mean of 22 weeks, whereas non-union was seen in only 4% (SR 9%, HR 0%). The overall complication rate was 36%. There was a significant correlation between the development of mechanical complications (fracture, delayed-/non-union) and a defect size of ≥12 cm (*p* = 0.013), segmental defects (*p* = 0.013) and additional required treatment (*p* = 0.008).

The use of non-vascularised fibulae dates back to the beginning of the twentieth century [23], but this technique has increasingly faded into the background after Taylor’s first description of a vascularised fibula reconstruction [24], as vascularised bone grafts were said to have a higher potential for hypertrophy and/or remodelling [25–27].

However, reports in the literature have been controversial. Hypertrophy rates for vascularised fibula reconstructions vary between 37% and 90% [1, 12, 15, 21, 28]. Additionally, significant differences between the hypertrophy rates at the upper and lower extremities have been described. For example, Hsu et al. reported a hypertrophy rate of 75% at the lower limb but only one out of seven upper limbs experiencing hypertrophy, which was attributed to the lack of mechanical forces at the upper limb [12]. In contrast, a series by Hilven et al. describe hypertrophy rates of 100% at the upper extremities and 86% in the lower limbs, which were assumed to be associated with the longer period of load restriction at the lower extremities. Likewise, the ability of non-vascularised fibula reconstructions to undergo hypertrophy at the host site has been controversially discussed. It was shown that non-vascularised grafts are inferior with regard to integration, resistance to bacterial infection and hypertrophy compared to vascularised grafts [29]. Nevertheless, there is evidence in the literature that even non-vascularised bone grafts are capable of remodelling and integrating into the host bone [13, 18, 20, 30]. On one hand, this might be constituted as a creeping substitution with viable cells migrating from the well-perfused conjunction zone into the vascular graft. On the other hand, the integration of avascular grafts could be attributed to a periosteal hypertrophy leading to new bone formation around the graft and eventual bony integration of the graft in some cases [21]. Therefore, we evaluated the presence and extent of hypertrophy at the graft-host junctions. In our series, hypertrophy was recorded in 85% of the evaluated graft-host junctions, and 52% of these hypertrophies were significant (>20%). No statistically significant differences were found between the upper and the lower extremities. Furthermore, our results were comparable or even superior to those at the pelvis (67% hypertrophy) despite the good soft tissue coverage and blood supply in the pelvic region [18].

In our series, primary consolidation (defined as consolidation within 12 months after surgery) was seen in 94% (SR 85%, HR 100%) of the host-graft junctions, with delayed union in 2% (SR 4%, HR 0%) and non-union in only 4% (SR 9%, HR 0%) of the patients. This is markedly superior to reports by Enneking and Yadav, who described primary union rates (within 12 months) of 63% and 60% for non-vascularised fibula grafts at the extremities [31, 32]. Likewise, Schuh et al. reported a union rate (defined as trabecular bridging within 6 months after surgery) of 67% for non-vascularised fibulae (non-union rate of 33%) and 85% (non-union rate of 15%) for vascularised grafts [20]. Based on the criteria of Schuh et al., we would have achieved union in 70% of cases (SR 60%, HR 85%) [20]. However, in our series, more than half of the patients (58%) underwent hemicortical reconstructions (Fig. 2), which have presumably higher consolidation rates due to the larger contact area as well as the lower extent of soft tissue dissection [7]. Similar to our results, comparable studies on hemicortical reconstructions with auto- or allografts following tumour resection showed lower non-union rates of only 0–7% [7, 33–36].

The application of additional treatment modalities such as chemotherapy might be one factor that contributes to the prolonged time to union. Hariri et al. reported a mean union-time of 1.75 years using vascularised fibula grafts, but all patients received neo-/adjuvant chemotherapy [37]. In our series, a total of 6 patients received neo-/adjuvant therapy (Table 1), and three of these patients had a delayed union or non-union. Though the administration of neo-/adjuvant therapy did not significantly influence the union time (*p* = 0.58), a statistically significant correlation between additional treatment and delayed union or non-union (*p* = 0.003) as well as the development of mechanical complications (*p* = 0.006) was observed.

In our series, patients with plate fixation had a longer consolidation time (mean 27 weeks) compared to patients with screw/press-fit fixation (mean 20 weeks). However, this difference was not statistically significant (*p* = 0.234). We have the opinion that the differences in the consolidation time might have been a problem of insufficient primary stability rather than of the plate fixation itself. Independently from the fixation technique, the defect size was a main factor that influenced the union time as there was a highly significant correlation between defect size and union-time (*p* < 0.001, R^2^ = 0.35). Additionally, a defect size of 12 cm or greater (*p* = 0.013) as well as segmental reconstructions (*p* = 0.013) were statistically significant risk factors for suffering a mechanical complication. Thus, 4 of our 5 fatigue fractures occurred in single strut reconstructions and bone defects of 12 cm or more, 3 of which were segmental defects. This is in accordance with reports in the literature, where the superiority of vascularised fibulae over non-vascularised grafts was reported for bone defects longer than 12 cm as indicated by failure rates of 25% and 50%, respectively, [38]. The significantly lower mechanical complication rate of hemicortical reconstructions (*p* = 0.013) in our series might be explained—beside the above mentioned factors such as the limited extent of soft-tissue dissection and a greater contact surface between graft and host bone—by the preservation of cortical continuity [7]. Taking our own results into account, we therefore strongly recommend the use of vascularised fibula grafts for segmental bone defects of 12 cm or greater.

In patients who were included in our previous study [13], no complications were recorded during the last 8 years (after the end of the previous study). Thus, the overall complication rate was 36% (*n* = 13) in our patients with a mean follow up of 8.3 years (range 2.1–26.6 years), among this subset, 77% (*n* = 10) needed revision surgery. The revision rate in the study by Schuh et al. was slightly higher at 48% of non-vascularised fibula reconstructions and 73% of vascularised fibula grafts [20]. Interestingly, the use of vascularised fibula grafts, a short graft-length and a lower extremity were shown to be risk factors for revision [20]. Likewise, Hariri et al. reported on a mean re-operative rate of 2.02 per patient after reconstruction with vascularised fibula grafts and an infection rate of 16% [37]. The infection rate in our study was only 6% (*n* = 2), one of which was superficial. For alternative treatment options such as intercalary allograft reconstructions, the incidence of complications varies from 7.5–30% for infections and 30–63% for non-union or delayed unions [3, 6, 39]. In diaphyseal tumour endoprostheses, failure rates of up to 63% at 10 years have been published, and patients generally contend with a life-long risk for complications such as infection [3, 40].

One shortcoming of autologous fibula grafts is the risk of donor site complications such as peroneal nerve palsy, stress fractures or joint instability [18, 37]. In our series, the donor site morbidity was rather low (6%) compared to those reported for vascularised fibula grafts (7–36%) [15, 37, 41, 42]. Additionally, we believe that this risk is acceptable, at least in non-vascularised fibula reconstructions which offer the advantage of remodelling capabilities at the host site as well as a technically less demanding surgical technique. In contrast to vascularised fibula reconstructions, tibial stress fractures haven’t been reported for non-vascularised fibula grafts until now, which might be attributed to the remodelling capacity at the donor site. Thus, among the analysed cases, complete remodelling was seen in 44%, partial in 40% and no remodelling in only 16% of the cases. In accordance with Grzegirzewski et al., all patients younger than 12 years showed complete remodelling, and patients presenting no remodelling were all older than 29 years [43].

If insufficient bone stock is preserved at the distal fibula, the risk for instability and valgus deformity of the ankle is high, especially in children [44]. In our series, in which at least 8 cm was preserved at the distal tibia and 4–5 cm at the proximal end, neither instability nor deformity was recorded at the knee or ankle joint. Two patients (6%) suffered a transient peroneal nerve palsy which recovered completely over time. The functional outcome of our patients was appealing and comparable to alternative treatment options as indicated by the mean MSTS score of 86%. Likewise, MSTS scores between 78% and 92% have been reported for vascularised fibula grafts [20, 37] and between 84% and 90% for diaphyseal tumour endoprostheses [2, 45].

The study’s retrospective design, small sample size, and lack of control groups are its main limitations. Our cohort was somewhat heterogeneous with respect to tumour entities, resection technique (segmental vs. hemicortical), use of additional treatments, age and lesion localization. The study period duration was extensive, but there were no changes over time regarding the surgical technique for this procedure, and only 2 surgeons performed all the operations. However, to our knowledge, this is the largest series of non-vascularised fibula reconstructions that has been published to date.

## Conclusions

The observed results regarding functional outcome, complications and consolidation of non-vascularised fibula reconstructions were encouraging, outside of the important advantage of this technique with respect to its remodelling capacity at the donor site. We therefore are of the opinion that the use of non-vascularised fibula grafts serves as a considerable alternative for bone defect reconstruction following resection of benign or early stage malignant tumours at the extremities. The application of this method is especially recommended for hemicortical reconstructions or patients with segmental defects of less than 12 cm in which no additional neo−/adjuvant treatment is to be administered.

## Abbreviations

Fig: Fig.
HR: Hemicortical reconstruction
MSTS: Musculoskeletal Tumor Society
SD: Standard deviation
SR: Segmental reconstruction
